# Evolution of reproductive morphology among recently diverged taxa in the *Drosophila mojavensis* species cluster

**DOI:** 10.1002/ece3.93

**Published:** 2012-02

**Authors:** Maxi Polihronakis Richmond, Sarah Johnson, Therese A Markow

**Affiliations:** Division of Biological Sciences, Section of Cell and Developmental Biology, University of CaliforniaSan Diego, La Jolla, California 92093

**Keywords:** Genitalia, morphological evolution, morphometrics, Sonoran Desert

## Abstract

The morphological evolution of sexual traits informs studies of speciation due to the potential role of these characters in reproductive isolation. In the current study, we quantified and compared genitalic variation within the *Drosophila mojavensis* species cluster to infer the mode of evolution of the male aedeagus. This system is ideal for such studies due to the opportunity to test and compare levels of variation along a divergence continuum at various taxonomic levels within the group. Shape variation was quantified using elliptic Fourier descriptors and compared among the four *D. mojavensis* host races, and between *D. mojavensis* and its sister species *Drosophila arizonae*. Aedeagus shape was diagnostic for *D. arizonae*, and among three of the four *D. mojavensis* subspecies. In each of these cases, there was less variation within subspecies than among subspecies, which is consistent with the pattern predicted if genitalia are evolving according to a punctuated change model, and are involved with mate recognition. However, aedeagus shape in *Drosophila mojavensis sonorensis* was highly variable and broadly overlapping with the other three subspecies, suggesting aedeagus evolution in this subspecies is more complex and subject to additional evolutionary factors. These results are interpreted and discussed in the context of selection on mate recognition systems and the potential for failed copulation.

## Introduction

The morphological evolution of sexual traits informs studies of speciation due to the potential role of these characters in reproductive isolation. This association, in combination with the breadth and scope of reproductive character diversity, has piqued interest in the evolution of reproductive morphology and its significance in population divergence. Several studies have shown sexual traits undergo dramatic shifts during speciation events (McPeek et al. 2008, 2009) due to alternating pressures arising from directional and stabilizing selection on mate recognition systems. Alternatively, sexual selection hypotheses posit that continuous variation within populations can accumulate over time and traits can diverge as a result of runaway processes or drift (Fisher 1930; Andersson 1994). While the outcome of both processes is the same, phenotypic divergence of sexual traits as a result of directional selection, the underlying dynamics of morphological evolution and population divergence are different.

Punctuated evolution resulting from selection on mate recognition has been documented in several systems where females frequently encounter heterospecific mates (reviewed in Gröning and Hochkirch 2008; McPeek et al. 2008). This process would necessarily result in reduced phenotypic variation of reproductive characters within populations due to stabilizing selection, and discrete variation among populations as a result of directional selection during speciation events (McPeek and Gavrilets 2006; Arbuthnott et al. 2010). On the other hand, sexual selection hypotheses predict that continuous selection on reproductive traits would result in high variation within populations reflecting patterns seen among populations (Arnqvist and Rowe 2002). While both hypotheses invoke directional selection as the catalyst of phenotypic change, the timing, duration, and strength of this selection can lead to dynamic outcomes (Boake et al. 1997; Hosken and Stockley 2004). Furthermore, both scenarios are subject to multiple indirect variables such as encounter frequency of heterospecific mates, population densities (Gowaty and Hubbell 2009), and resource competition that may mask our ability to disentangle the processes influencing morphological change. One way to test these hypotheses and investigate how sexual traits evolve is to quantify levels of variation within and among incipient species.

**Figure 1:**
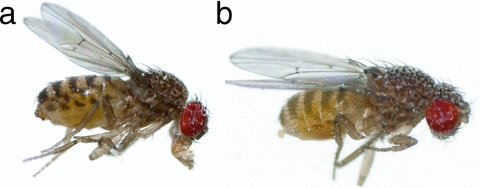
(a) *Drosophila arizonae* (with mouthparts extended), (b) Drosophila mojavensis. Specimens can often be distinguished by the pattern of markings on the lateral tergites. In *D. arizonae*, the pattern consists of more obvious triangular shapes. Photo courtesy of Luciano Matzkin.

**Figure 2:**
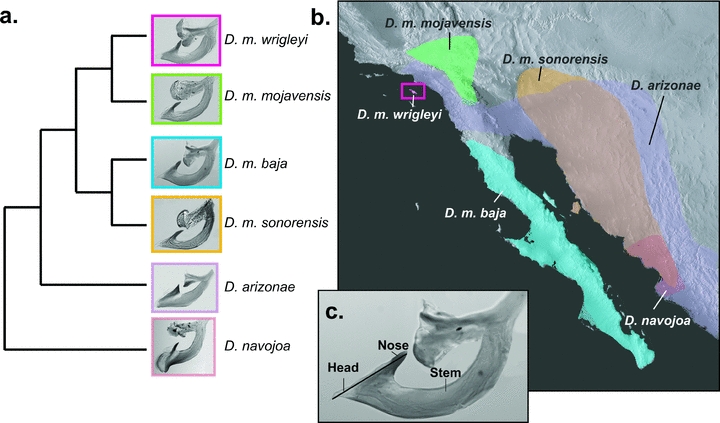
(a) Phylogenetic relationships of the *Drosophila mojavensis* species cluster (fromMachado et al. 2007) with images of the male aedeagus for each group, (b) corresponding geographic distribution for each taxon in the phylogeny, (c) image of aedeagus illustrating regions discussed in the text: “head” (including measurement line for genitalia size), “nose,” and “stem.”

The *Drosophila mojavensis* species cluster presents an optimal combination of taxa in which to examine the morphological evolution of sexual traits. It is comprised of three species of cactophilic drosophilids; *D. mojavensis* (Fig. 1a), *D. arizonae* (Fig. 1b), and *Drosophila navojoa*, all found in the desert regions of North America (Fig. 2). Furthermore, *D. mojavensis* is split into four subspecies based on phylogenetic and population genetic analyses of geographically isolated populations that specialize on different host cacti (Machado et al. 2007; Reed et al. 2006; Pfeiler et al. 2009). Each species occupies a unique ecological niche with *D. arizonae* feeding primarily on the columnar cactus *Stenocereus alamosensis*, but able to utilize a variety of other cactus species, as well as citrus (Pfeiler et al. 2009). On the other hand, the four *D. mojavensis* subspecies each specialize on different cacti in their respective geographic regions. A recent taxonomic treatment of *D. mojavensis* provided subspecies accounts for the four subspecies; *Drosophila mojavensis wrigleyi*, *Drosophila mojavensis baja*, *Drosophila mojavensis sonorensis*, and *Drosophila mojavensis mojavensis*, and clarified the evidence supporting their distinction (Pfeiler et al. 2009). Studies quantifying the extent of prezygotic reproductive isolation between species in the *D. mojavensis* species group, as well as among the four *D. mojavensis* subspecies, have revealed an intriguing mosaic of outcomes depending on which populations of a particular sex are involved (Wasserman and Koepfer 1977; Ruiz et al. 1990; Markow 1991; Reed and Markow 2004; Massie 2006). This mosaic represents a complex continuum of incipient speciation that is ideal for identifying the chronology of reproductive isolation accompanying ecological host shifts, as well as the corresponding divergence of morphological characters.

Interestingly, however, despite strong evidence that genitalic morphology plays a large role in reproduction isolation among these taxa (Markow 1981; Krebs and Markow 1989; Hocutt 2000), the genitalic variation described among the four subspecies (Pfeiler et al. 2009) remains uninvestigated in the context of sexual selection and speciation. We thus undertook an in-depth quantitative analysis of genitalic shape variation within and among the four *D. mojavensis* subspecies and *D. arizonae. Drosophila navojoa*, the sister species of *D. mojavensis* and *D. arizonae*, was not included because the structure and shape of their genitalia are markedly different, and there is very little evidence to support hybridization, even in the laboratory. Specifically, we analyzed the shape of the male aedeagus to address the following questions: (1) Can shape predict species and subspecies identity? (2) What is the relative trajectory of shape change between pairwise comparisons of *D. mojavensis* subspecies? (3) What do size and shape variation patterns within and among species tell us about the mode of evolution of this character? (4) Can we make predictions about morphologically based isolating mechanisms based upon the shape differences we uncover?

## Methods

*Fly strains:* We used laboratory strains of each of the four *D. mojavensis* subspecies and one strain of *D. arizonae* (Table 1). In addition, wild males were collected of *D. m. sonorensis* (Las Bocas, Sonora, just 30 km north of the Agiabampo stock collection site) and *D. arizonae* (from Tucson, as was the laboratory stock) in order to compare the influence of field and laboratory environments on aedeagus shape.

**Table 1:** Lab stocks used from the Drosophila Species Stock Center (DSSC) at UC San Diego with corresponding collection data

Species	DSSC stock number	Locality (year collected)
D. arizonae*	15081–1271.18	Tucson, AZ, USA (2004)
*D. m. baja*	15081–1351.30	Punta Prieta, Baja California, Mexico (2008)
*D. m. mojavensis*	15081–1352.01	Anza Borrego Desert, CA, USA
*D. m. sonorensis*	15081–1352.26	Agiabampo Bay, Sonora, Mexico (2003)
D. m. wrigleyi*	15081–1352.02	Catalina Island, CA, USA (1991)

* Stocks used in reciprocal hybrid crosses.

*Culture and handling of flies:* All laboratory rearing was performed on banana medium at 23°C. Two replicates were set up for each species and subspecies, and consisted of crosses between 10 mature virgin male and female flies in half pint bottles. After four days, adults were transferred to new bottles, and discarded four days later. Upon emergence, F1 adults were removed every 24 h and allowed to mature for five days before being preserved in 70% ethanol. Thus, all adults used for genitalic dissections were considered virgin. Twenty adults from each *D. mojavensis* subspecies and *D. arizonae* were randomly chosen from each replicate for analysis. Analysis of the F1 hybrids included 10 adults from each cross. To compare patterns of genitalic variation of lab stocks versus wild populations, we included 20 *D. m. sonorensis* males collected in Las Bocas, Sonora, Mexico, and 10 *D. arizonae* males collected in Tucson, AZ. For each dissection, thorax length was measured and adults were placed in warm KOH (pH 10) for 30 min before removal of the male aedeagus. The aedeagus was mounted on a microscope slide and a lateral view was imaged at 400× using a Nikon Eclipse E800 compound microscope fitted with a RT Monochrome camera (Diagnostic Instruments, Inc., Sterling Heights, Michigan, USA).

Images were edited in Adobe Photoshop CS2 (Adobe Systems, Inc., San Jose, California, USA) to create black and white outline files for analysis in the program SHAPE v1.3 (Iwata and Ukai 2002). In brief, this program uses two-dimensional images, in this case a lateral view of the aedeagus, to calculate principal component (PC) scores based on a variance–covariance matrix created from the coefficients of the elliptic Fourier descriptors using 20 harmonics. These PCs are considered comparable to continuous measurements of morphological characters and can be used as such in subsequent statistical analyses. Because the Fourier coefficients are normalized based on the first harmonic ellipse, size is factored out of the PC analysis.

Variation of the male aedeagus was analyzed in SHAPE in four separate comparative analyses, (1) *D. arizonae* plus all *D. mojavensis*, (2) the four *D. mojavensis* subspecies, (3) the F1 hybrids plus the two parental strains (*D. arizonae* and *D. m. wrigleyi*, and (4) *D. m. sonorensis* and *D. arizonae* lab stock males versus wild-caught males. For all four analyses, the first two significant PCs were plotted against each other to visualize the relative morphological space for the different comparisons. For the second analysis including all *D. mojavensis* individuals, we tested whether the shape variation described by the PCs was significantly different among the subspecies using an analysis of variance (ANOVA) on the effective PCs in JMP 9.0.0 (SAS Institute Inc., Cary, North Carolina, USA). Variation within populations was compared by testing for equal variance among all pairwise comparisons of each *D. mojavensis* subspecies using a Levene's test in JMP 9.0.0. Goodness-of-fit tests were done in JMP 9.0.0 on significant PCs to test for deviation from a normal distribution. In order to assess the trajectory of shape evolution in each of the four *D. mojavensis* subspecies, SHAPE analyses were conducted on all possible pairwise subspecies comparisons. These analyses provide a means to compare shape changes occurring between each subspecies pair. Lastly, we performed a discriminant function analysis of the *D. mojavensis* dataset to test how well individuals could be assigned to subspecies using the significant PC scores.

Patterns of genitalia and body size variation were analyzed in conjunction with genitalic shape. Genitalia size was measured in Photoshop by drawing a line with the measurement tool along the longest axis of the “head” portion (see Fig. 2c). To test whether genitalia and body size were significantly different among the *D. mojavensis* subspecies, an ANOVA was performed on genitalia and body size measurements in addition to a post hoc comparison of means using Tukey's HSD test. Equal variance of genitalia and body size among subspecies was analyzed using a Levene's test. Genital allometry was assessed by regressing genitalia size against body size within each subspecies, and for all *D. mojavensis* individuals combined.

## Results

### Inter- and intraspecific variation in aedeagus shape

**Figure 3:**
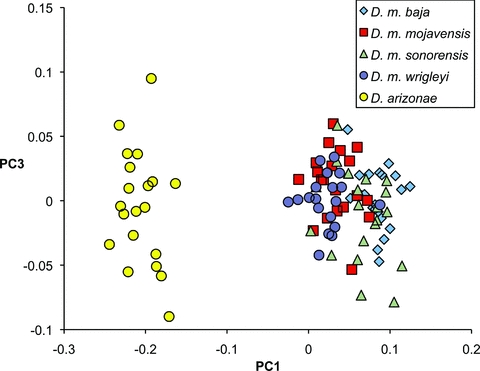
Morphological shape space of *Drosophila arizonae* and Drosophila mojavensis.

Seven effective PC scores for the analysis including *D. arizonae* and the four *D. mojavensis* subspecies described 95.88% of the total variation (Table 2).Figure 3 provides a visualization of the morphological space of *D. arizonae* relative to *D. mojavensis*, and reveals no overlap between *D. arizonae*, and that of any of the four *D. mojavensis* subspecies.

**Table 2:** Shape analysis 1 (D. mojavensis and *D. arizonae*); seven effective principal components (PCs), corresponding eigenvalues, and proportion of variance explained

PC	Eigenvalue (×10^−2^)	Proportion of variance (%)
1	1.1797	75.00
2	0.1088	6.92
3	0.0989	6.35
4	0.0501	3.19
5	0.0253	1.61
6	0.0237	1.51
7	0.0205	1.30
Cumulative variance (%)		95.88

**Figure 4:**
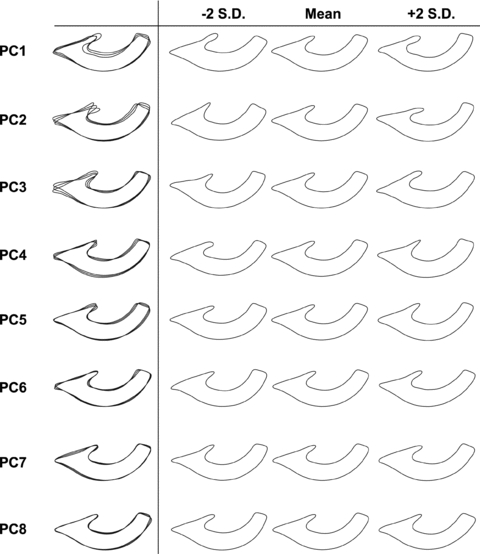
Contour reconstruction of aedeagus shape variation among the four *Drosophila mojavensis* subspecies described by each effective principal component (PC). As described in the text, overlapping lines in the first column denote areas of aedeagus variation. The remaining three columns provide a nonoverlapping visualization of these same contours.

In the shape analysis including all four *D. mojavensis* subspecies, eight effective PCs described 93.35% of the total variation (Table 3). The ANOVA revealed that PC 1 and PCs 3–6 were significantly more variable among subspecies than within (Appendix 1). A visualization of the shape variation of the lateral view of the aedeagus described by the eight effective PCs is illustrated in the contour reconstruction of the mean and standard deviation (Fig. 4). Nonoverlapping lines in the first column represent variation of the aedeagus reflected by the respective PC. Thus, based on these contours, it is possible to visualize what shapes are significantly different among groups (PC1 and PC3–6) relative to what shapes are not (PC2, PC7-8). Much of the shape variation described by PC1 occurs in the region of the head. The largest discrepancy is in the length of the “nose,” and the degree of notching underneath the nose where it connects to the stem. Variation described by PC1 can also be seen in the width at the tip of the head region as well as in the curvature underneath where it connects to the stem. In addition to variation of head shape, PC1 also describes variation associated with the width of the stem, which appears to be related to the degree of notching underneath the nose. For PC2, the variation illustrated that is not significantly different among subspecies is largely associated with the degree of rotation of the head, in addition to the width of the most basal portion of the stem. Interestingly, variation in head rotation is also seen in PC3, which is significantly different among subspecies, but appears to co-vary less with the position of the nose as seen in PC2. Last, PC3 describes variation in the width of the nose, and the degree of notching underneath the nose.

**Table 3:** Shape analysis 2 (four D. mojavensis subspecies); eight effective principal components (PCs), corresponding eigenvalues, and proportion of variance explained

PC	Eigenvalue (×10^−2^)	Proportion of variance (%)
1	0.1966	39.95
2	0.1059	21.52
3	0.0766	15.56
4	0.0292	5.94
5	0.0199	4.04
6	0.0133	2.69
7	0.0104	2.11
8	0.0075	1.53
Cumulative variance (%)		93.35

The Levene's test of equal variance within each subspecies revealed that *D. m. sonorensis* exceeded the upper limit for both PC1 and PC3. Thus, genitalic shape of this subspecies is significantly more variable than the other three subspecies. Because this result violates the assumption of equal variance for the ANOVA, we evaluated the affect of this result by running the ANOVA with and without *D. m. sonorensis*. The results from both analyses were significant. Goodness-of-fit tests on PC1 and PC3 could not reject the hypothesis that these data were normally distributed.

### Prediction of subspecies by aedeagus shape

**Figure 5:**
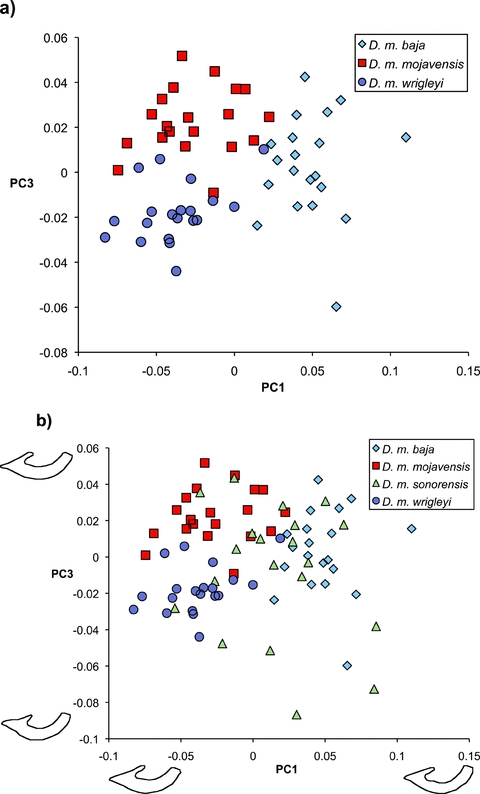
(a) Morphological shape space *D. m. baja, D. m. sonorensis,* and *D. m. wrigleyi*, (b) morphological shape space of all four *D. mojavensis* subspecies.

Figure 5 illustrates the morphological space and discrete differences in aedeagus shape variation among three of the four *D. mojavensis* subspecies. When PC1 is plotted against PC3, there is little to no overlap between *D. m. baja* and *D. m. mojavensis*, and *D. m. baja* and *D. m. wrigleyi* (Fig. 5a). However, *D. m. sonorensis* overlaps the morphological space of the three other subspecies (Fig. 5b).

**Figure 6:**
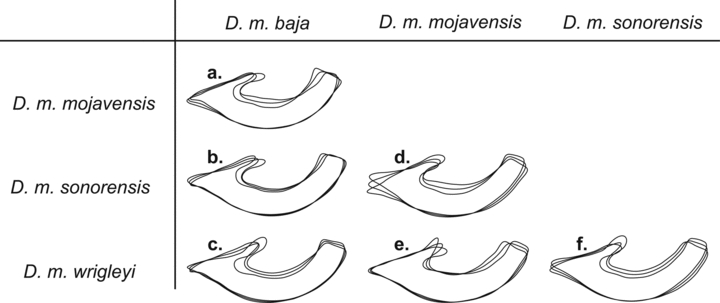
Pairwise comparison of PC1 shape variation for all possible combinations of the four *Drosophila mojavensis* subspecies.

Pairwise comparisons of PC1 provide a direct visualization of the type of shape changes occurring between each pair of subspecies (Fig. 6). As in the contours in Figure 4, areas where the lines do not overlap represent the variation described by a particular PC, in this case PC1. It is apparent fromFigure 6 that shape evolution of the aedeagus in each subspecies is occurring along a different trajectory. For example, comparison of pairwise shape changes between *D. m. baja* and *D. m. mojavensis* (Fig. 6a), and *D. m. wrigleyi* and *D. m. mojavensis* (Fig. 6e) illustrates how the former pair varies most in the length of the head region while the latter varies most in the angle of the nose. Similarly, the majority of shape variation between *D. m. baja* and *D. m. wrigleyi* (Fig. 6c) occurs in the length of the head as well as in the notch between the stem and head region, while that between *D. m. sonorensis* and *D. m. wrigleyi* (Fig. 6f) is most evident in the roundness and length of the nose. On the other hand, comparisons between *D. m. baja*, *D. m. mojavensis*, and *D. m. sonorensis* (Fig. 6a and 6b) reveal a higher degree of similarity in the types of changes occurring between these pairs.

The discriminant function analysis correctly classified 86% of the individuals to their subspecies designation. Of the 11 individuals that were misclassified, seven of those were *D. m. sonorensis*. Interestingly, the predicted assignment for the misclassified *D. m. sonorensis* included each of the three other subspecies suggesting affinity not just to one subspecies, but to all. Further, with only one exception, the other four individuals of the other subspecies that were misclassified were predicted to be *D. m. sonorensis* (two *D. m. baja* and one *D. m. wrigleyi*). The exception was one *D. m. mojavensis* that was predicted to belong to *D. m. baja*. Thus, male genitalic shape appears to be diagnostic in three of the four *D. mojavensis* subspecies.

### Allometry of genitalia and body size

**Figure 7:**
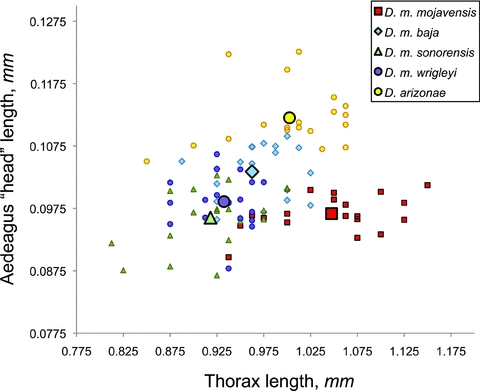
Body size versus genitalia size. Large symbols denote averages for each taxonomic group.

The ANOVA of body size and genitalia size revealed significantly more variation among subspecies than within subspecies (Appendix 2). Comparison of means for body size using Tukey's HSD test revealed that all comparisons among subspecies were significantly different with the exception of *D. m. wrigelyi* and *D. m. baja*, and *D. m. wrigleyi* and *D. m. sonorensis*. The largest subspecies was *D. m. mojavensis* and the smallest was *D. m. sonorensis* (Fig. 7). In contrast, the comparison of means for genitalia size revealed that *D. m. baja* had significantly larger genitalia than the other subspecies (Fig. 7). Differences in genitalia size among the other three subspecies were not significant. The results of the Levene's test of equal variance for genitalia and body size showed that mean absolute deviations from the median (ADM) were within the upper and lower bounds. This is in contrast to genitalia shape where *D. m. sonorenesis* exceeded the upper bound for PC1 and PC3.

There was no significant correlation between genitalia and body size when each subspecies was tested individually, or when all *D. mojavensis* were plotted together. Body size to genitalia size ratios for each subspecies were as follows: *D. m. baja*, 5.18:1; *D. m. mojavensis*, 4.47:1; *D. m. sonorensis*, 5.07:1; *D. m. wrigleyi*, 5.13:1 (*D. arizonae*: 5.4:1).

### Shape variation of *D. mojavensis*×*D. arizonae* F1 hybrids

**Figure 8:**
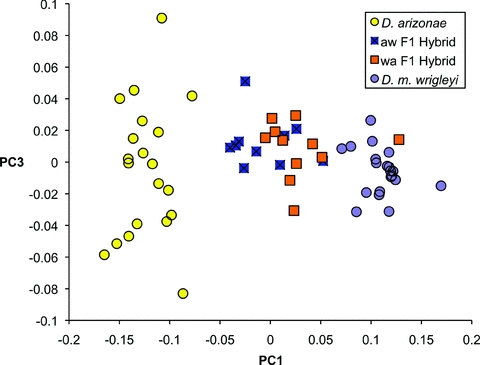
Morphological shape space of F1 hybrids from reciprocal crosses with the two parental lines.

Analysis of shape variation between reciprocal F1 hybrids resulted in four effective PCs describing 93.4% of the variation. The plots of PC1 against PC3 of the reciprocal hybrids and two parental lines are illustrated in Figure 8. Interestingly, both sets of F1 hybrids occupy overlapping morphological space between the two parental lines, and only the means of PC1 are significantly different (*t* = 2.50, *P* = 0.0108). While both sets of F1 hybrids were intermediate between the two parental lines, when the mother was *D. m. wrigleyi*, the aedeagus of the sons was more similar to the males of the maternal line. Likewise, when the mother was *D. arizonae*, aedeagus shape was more similar to that of *D. arizonae* males (Fig. 8).

### Shape variation of lab stocks versus wild caught males

Aedeagus shape was largely uninfluenced by rearing environment in either species. There was no significant difference between the *D. m. sonorensis* lab stock males and wild caught males for PCs 1, 3, 4, and 5. While PC2 was significant, this could reflect some slight regional variation. In the analysis comparing *D. arizonae* lab stock and wild caught males all collected from Tucson, AZ, none of the PCs were significant. Females of both species mating in nature are therefore exposed to the same genitalic shapes as in the laboratory.

## Discussion

Investigation into the patterns of shape variation in male genitalia of the *D. mojavensis* species cluster revealed significant shape differences between *D. mojavensis* and *D. arizonae*, and among three of the four *D. mojavensis* subspecies. For *D. m. baja*, *D. m. mojavensis*, and *D. m. wrigleyi*, shape variation was diagnostic and accurately predicted subspecies identity in a discriminant function analysis. In addition, the degree of shape variation within each of these subspecies was comparable and did not exceed the upper bound of ADM. This is the pattern predicted if genitalia are evolving according to a punctuated change model with most change occurring at the time of divergence as a result of selection for mate recognition (McPeek et al. 2008, 2009). Alternatively, aedeagus shape in *D. m. sonorensis* was highly variable and broadly overlapping with the other three subspecies, suggesting the mode of aedeagus evolution is not the same for all four *D. mojavensis* subspecies.

### Aedeagus shape predicts species and subspecies identity

*Drosophila arizonae* and *D. mojavensis* exhibited significant differences in both genitalic shape and size. The aedeagus of *D. arizonae* was larger and more robust than that of *D. mojavensis*. Both the stem and head regions were thicker with a shorter pointed nose region (Fig. 2c). These two sibling species of cactophilic drosophilids are predicted to have diverged approximately 0.5 million years ago (mya) based on nuclear data (Matzkin et al. 2005), although mitochondrial data pushes the split back to 1.91–2.97 mya (Reed et al. 2006). Both species are broadly represented in the Sonoran Desert with *D. arizonae* continuously distributed throughout the region while *D. mojavensis* occupies four distinct geographic areas (Fig. 2b). Sympatry of *D. arizonae* and *D. mojavensis* occurs in mainland Mexico in the southern portion of the range of *D. m. sonorensis*, as well in Anza Borrego Desert where *D. m. mojavensis* is found. Recent collections of *D. arizonae* in the Baja California peninsula suggest new zones of sympatry with *D. m. baja*. While there is no evidence to support hybridization in localities where these two species are sympatric (Machado et al. 2007), varying degrees of hybridization will occur in the lab depending on which populations are crossed (Wasserman and Koepfer 1977; Ruiz et al. 1990; Reed and Markow 2004; Massie 2006).

Discrete shape variation among *D. m. baja*, *D. m. mojavensis*, and *D. m. wrigleyi* provides further support for the subspecies designations presented byPfeiler et al. (2009). Each subspecies occupied a unique position in morphological shape space, and was less variable than the species as a whole. Diagnostic morphological variation of aedeagus shape is consistent with previous ecological, genetic, and behavioral datasets supporting reproductive isolation among these geographically isolated subspecies (Pfeiler et al. 2009 and refs. therein). The pairwise comparisons of shape change were consistent with this result and revealed variation in the types of shape changes occurring between species. While some pairs, such as *D. m. baja* and *D. m. mojavensis* varied in the length of the head region and width of the tip; others, such as *D. m. baja* and *D. m. wrigleyi*, differed in the roundness of the nose, and notch underneath the head.

Aedeagus shape in *D. m. sonorensis* was not diagnostic, and overlapped with each of the other three subspecies in the plot of morphological shape space (Fig. 5). In the discriminant function analysis, *D. m. sonorensis* specimens could not be accurately classified and were assigned to each of the other three subspecies. Lastly, within-population variation of *D. m. sonorensis* aedeagus shape was significantly higher than in the other three subspecies. When considered all together, these patterns suggest that the mode of aedeagus shape evolution in *D. m. sonorensis* is not the same as in the other subspecies. Review of previous work on this system reveals two hypotheses that could explain the differential divergence patterns observed. Sympatry between *D. m. sonorensis* and its sister species, *D. arizonae*, could result in stronger selection pressures on pre-copulatory aspects of the mate recognition system rather than post-copulatory. Alternatively, gene flow across the Sea of Cortez in an east-west direction (in concordance with weather patterns) could lead to gene flow from *D. m. baja* in the peninsula to *D. m. sonorensis* in mainland Mexico. These hypotheses, discussed in more detail below, are not necessarily mutually exclusive and may both contribute to the observed patterns of aedeagus evolution in *D. m. sonorensis*.

Pre-copulatory mate recognition is hypothesized to play a stronger role in reproductive isolation where *D. mojavensis* subspecies are sympatric with *D. arizonae*. For example, previous studies have shown that *D. m. sonorensis* females are more selective in choosing mates than *D. m. baja* females (Wasserman and Koepfer 1977; Zouros and Dentremont 1980), while *D. m. sonorenis* males were less selective (Krebs and Markow 1989). The patterns of variation of *D. m. sonorensis* male genitalia provide further support for the hypothesis that reproductive isolation of *D. m. sonorensis* may be subject to different selection pressures relative to the other three subspecies due to its higher degree of sympatry with *D. arizonae*. Interestingly, the *D. m. sonorensis* individuals sampled in this study are the only ones that exist in sympatry with *D. arizonae*. However, new records of *D. arizonae* from the Baja peninsula suggest a recent range expansion, which may shift the evolutionary dynamics with regard to reproductive isolation and reinforcement, similar to patterns documented in other areas of sympatry. Corroboration of these results with experiments looking at additional sympatric subspecies combinations would provide further support for this hypothesis.

The second hypothesis to explain higher levels of aedeagus variation within *D. m. sonorensis* comes from evidence supporting gene flow with *D. m. baja* coming across the Sea of Cortez (Reed et al. 2006). Population genetic analyses using the mitochondrial COI gene revealed shared haplotypes between several *D. m. sonorensis* and *D. m. baja* individuals. Increased genetic heterogeneity on the mainland could parallel higher heterogeneity of morphological structures.

Our study also detected differences between the subspecies based on size of genitalia relative to body size. *D. m. wrigleyi* and *D. m. sonorensis* were the smallest flies, and had the smallest genitalia. Alternatively, *D. m. mojavensis* was the largest fly but had comparably sized genitalia to *D. m. wrigleyi* and *D. m. sonorensis*. Last, *D. m. baja* was smaller than *D. m. mojavensis* but had the largest genitalia. Regardless of the relative differences in body and genitalia size among the four subspecies, there was no evidence for positive allometry when each of the subspecies were tested individually, or when all four were analyzed together. This result is consistent with the one-size-fits-all hypothesis explaining the general pattern of negative static allometry of arthropod male genitalia (Eberhard et al. 1998), but does not necessarily imply that these structures are under stabilizing selection (Eberhard et al. 2009).

### Mode of aedeagus shape evolution

While not all together surprising, the distinct shape variation between *D. arizonae* and *D. mojavensis* is intriguing because these species will hybridize in the lab. The degree of reproductive isolation in lab-based matings is influenced by whether parental lines are allopatric or sympatric (Markow 1981). Isolating mechanisms include precopulatory behavioral isolation as well as an unknown postcopulatory factor. In the latter case, which we refer to as pseudocopulation, females will accept male courtship attempts but copulation is not successful. Based on the discrete shape differences revealed in this study, future studies investigating how the mechanics of aedeagus shape affect copulation success would provide much-needed details regarding the role of pseudocopulation as an isolating barrier (Richmond et al. 2011). In addition, studies of pseudocopulation between *D. arizonae* and *D. mojavensis* would establish a baseline with which to compare similar data collected between *D. mojavensis* subspecies, which are less divergent in shape.

Based on the plots of aedeagus shape of F1 hybrids from reciprocal crosses between *D. arizonae* and *D. mojavensis*, it appears that aedeagus shape of F1 sons is more similar to aedeagus shape of the maternal line (Fig. 8). This would suggest that genes involved in determining genitalia shape are X-linked or maternal effects. Previous studies investigating the genetics of genitalia development have generated mixed results.Coyne et al. (1991) found no significant effect of the X chromosome on genitalia whileLiu et al. (1996) found a QTL on the X chromosome that explained 5.7% and 14.5% of the variation in PC1 for *D. mauritiana* and *D. simulans*, respectively. In a more recent study investigating the genetic basis of size and shape variation of the male genitalia in *D. sechellia* and *D. mauritiana*, Masly et al. (2011) identified a gene on the X chromosome (NENEH2(A)) that appeared to have directional effects on genital shape. While these studies focused on the external posterior lobe of the male genitalia, both the posterior lobe and the aedeagus develop from the genital disc and thus further investigation of such loci would likely yield important information about the genetic basis of shape variation of the aedeagus in *D. mojavensis*.

The amount of within subspecies shape variation for *D. m. baja*, *D. m. mojavensis*, and *D. m. wrigleyi* was significantly less than among subspecies variation, and did not exceed the upper bound in a Levene's test of equal variance. This pattern is consistent with the hypothesis that evolution of aedeagus shape results from oscillating bouts of stabilizing selection between divergence events, and directional selection at the time of speciation. This type of selection regime on reproductive characters supports a role for the aedeagus in mate recognition. Evidence for genitalia as a mate recognition tool is relatively rare due to the evolutionary significance of precopulatory mechanisms, such as behavior and courtship songs, which precede interaction of male and female reproductive morphology (Henry 1985; Arbuthnott et al. 2010). However, there are several documented cases from various arthropod groups including *D. simulans* and *D. mauritiana* (Coyne 1993), *Parafontaria* millipedes (Tanabe and Sota 2008), and *Enallagma* damselflies (McPeek et al. 2008). There is also evidence that copulatory structures play a role in mate recognition in *D. mojavensis* (Hocutt 2000). In a series of mate trials between *D. mojavensis* subspecies, there was significant variation in a male's ability to achieve the appropriate copulatory position after females agreed to mate. The resulting pseudocopulation was shorter in duration with little to no sperm transfer. As mentioned above, conducting additional pairwise comparisons of pseudocopulation among *D. mojavensis* subspecies presents a unique opportunity to identify aspects of aedeagus shape that influence reproductive isolation between taxonomic groups at various stages of differentiation. This would be particularly interesting in light of the results presented here that not all *D. mojavensis* subspecies are following the same evolutionary trajectory. Specifically, based on the pairwise comparisons of shape change between the *D. mojavensis* subspecies, it would be possible to test predictions regarding which types of shape changes were more likely to result in failed copulation attempts.

### Conclusions

The *D. mojavensis* species cluster is a model system for speciation studies and has provided a wealth of data on the processes of reproductive isolation in incipient species. In the current study, we provide an additional role for this system as a means to investigate the evolution of reproductive morphology. The continuum of divergence within this species cluster provides an ideal arena to test hypotheses explaining the influence of genital shape evolution on reproductive isolation. In the current study, we focused on identifying patterns of variation within and among the *D. mojavensis* species cluster to establish a foundation for generating testable predictions for subsequent studies on the mechanisms and outcomes of pseudocopulation. Understanding the mechanical restraints of morphology in recently diverged populations will provide a framework for identifying the role of morphological evolution in reproductive isolation.

## Appendix

**Table A1:** ANOVA of effective principal components (PCs) describing aedeagus shape variation among the four *Drosophila mojavensis* subspecies

Source of variation	Sum of squares	*F* ratio	Probability > *F*
PC1	0.0950	39.9315	<0.0001
PC2	0.0053	1.7185	0.1703
PC3	0.0179	10.6338	<0.0001
PC4	0.0105	21.3179	<0.0001
PC5	0.0028	5.4169	0.0020
PC6	0.0024	7.6540	0.0002
PC7	0.0004	1.2300	0.3047
PC8	0.0001	0.6436	0.5894

**Table A2:** ANOVA results for genitalia and body size among the four *Drosophila mojavensis* subspecies

Source of variation	Sum of squares	*F* ratio	Probability > *F*
Body size	3.2198	32.4994	<0.0001
Genitalia size	25.1477	13.9774	<0.0001
